# Supplementary material for: SDG5 “Gender Equality” and the COVID-19 pandemic: A rapid assessment of health system responses in selected upper-middle and high-income countries
Source: Front Public Health. 2023 Feb 3;11:1078008. doi: 10.3389/fpubh.2023.1078008 (PMC9935821; doi:10.3389/fpubh.2023.1078008)
Supplement: Supplementary Table S1 — Australia, country case study summary. [file Table_1.pdf]

Supplementary material. Table S1 Australia, country case study summary

SDG5	Impact	Action	Future Policy
**Maternity care/ reproductive health**			
Summary	essential services were kept open with some limitations and new digital services; restrictions to prevention and support services; evidence of increased rates of antenatal depression; some improvement in reproductive health services and rights	overall little attention but some expansion of reproductive health services, including early abortion; new digital antenatal services are included in Medicare Benefits Schedule (MBS)	some antenatal expansions were maintained; currently no decision on reproductive services and rights
Maternity care	access to essential services, but restrictions due to pandemic policies (face mask during birthing; no access of partners; etc.) with strong local variation; new digital services and strong expansion; restrictions in support and non-essential services	new digital antenatal services were added to healthcare delivery, covered by the MBS	new digital antenatal services included in the MBS
Reproductive services	essential services were kept open but pandemic restrictions on elective surgery included fertility treatments; some variation; early medical abortion was made available via digital consultation; choice of provider for reproductive health consultations	early medical abortion via digital appointments was introduced in the MBS; digital provision of reproductive health consultations was reimbursed by MBS and restriction on choice of provider was relaxed	currently no decision
Reproductive rights	some improvement through relaxed legal restrictions of early abortion and improved MBS rules on choice of provider	no legal action but some improvements through changes in the MBS scheme	currently no decision
Care for pregnant women/ vaccination	included in vaccination programs	since June 2021, pregnant women are included in prioritised groups	none
Health labour market and employment	higher infection risk of midwives and other HCWs; shortage may worsen	none	none
Social inequalities	lack of data; digital services provision may increase social inequality and affect vulnerable groups most strongly; employment loss during the pandemic may restrict fertility decisions	none	none
**Gender-based violence**			
Summary	increase but poor data; some attention to the problem and expansion of services; digital service provision	Government introduced new programs and significantly increased budgets; improved support for victims, some attention to men/offenders and violence protection	some attention and new programs
Access to services	routine health and legal services were kept open but often moved to digital provision; regional variation; digital services may restrict access	Coronavirus Domestic Violence Support Package introduced in March 2020 including AUS 150 million	some attention and new programs

		budget and National Partnership Agreement on COVID-19 Domestic and Family Violence Responses; responses to increased demand and new needs, e.g. supporting transformation to digital provision and people in remote/rural areas	
Scaling-up/ new programs and training programs	expansion of family violence phone services; expansion of support, e.g. short-term accommodation; improved protective equipment; improved technical support; support for men's behaviour programs; high regional variation; improved awareness but no new training programs to improve sensitivity	significant increase in budgets, including an increase in capacity of nationwide family violence services, men's counselling, perpetrator intervention, women's safety at home, support for trafficked people; in Victoria additional funding was made available to meet increased demand	introduced programs extended or new programs added
Access of HCWs to help-lines	some local health services may be available but no national program; high variation	None	none
Social inequalities	pandemic restrictions threaten vulnerable groups most strongly; digital provision exacerbates inequalities	lack of attention but some support for people in remote areas to mitigate disadvantages of digital services	none
**Gender equality**			
Summary	lack of attention to gender equality and mainstreaming policy; poor participation of women and female leadership; some support for HCWs with childcare responsibilities	none, except provision of free childcare during the first period of lockdowns	none
Women/ female leadership in health policy and COVID-19 governing boards	lack of attention to female leadership and participation in National Advisory Committee; overall lack of data	none; equal opportunity law not systematically applied to pandemic policy and governance; no monitoring	none
Gender equality goals in pandemic policy	None	None	none
Gender equality goals in research	lack of attention and incentives	None	none
Access to childcare	facilities were kept open with a few exemptions; regional variation	Government provided free childcare during lockdown in 2020; in case of closure services were kept open for HCWs and vulnerable families	none
Access to schools	periods of closures but strong regional variation; move to digital schooling	schools were kept open for essential workers/HCWs and vulnerable families	none
Social inequalities	increase in gendered inequalities with intersectional effects	lack of attention but some support for remote areas	none

Source: authors' table, based on expert information, published secondary sources

Key references

Australian College of Midwives (ACM). Women's experiences of maternity care at the height of COVID-19. Canberra: Australian College of Midwives (2020). Available online at: https://www.midwives.org.au/news/womens-experiences-maternity-care-height-covid-19 (accessed 2022, October 20).

Boxall H, Morgan A, Brown R. The prevalence of domestic violence among women during the COVID-19 Pandemic. Statistical Bulletin no. 28. Canberra: Australian Institute of Criminology (2020). Available online at: https://www.aic.gov.au/publications/sb/sb28 (accessed 2022, October 2022).

Department of Health and Human Services. Visiting hospitals: rest of Victoria Melbourne. Canberra: Australia Department of Health and Human Services (2020). Available online at: https://www.dhhs.vic.gov.au/visiting-hospitals-covid-192020 (accessed 2022, October 20).

Frankham LJ, Thorsteinsson EB, Bartik W. Antenatal depression and the experiences of Australian women in the maternity system during the COVID-19 pandemic. Open Journal of Depression. (2021) 10:155–167. doi:10.4236/ojd.2021.104010

Johnston RM, Mohammed A, van der Linden C. Evidence of exacerbated gender inequality in child care obligations in Canada and Australia during the COVID-19 pandemic. Politics & Gender. (2020) 16(4):1131–1141. doi:10.1017/S1743923X20000574

National Health and Medical Research Council. National COVID-19 Health and Research Advisory. Canberra: NHMRC (2021). Available online at: https://www.nhmrc.gov.au/about-us/leadership-and-governance/committees/national-covid-19-health-and-research-advisory-committee (accessed 2022, October 20).

Parliament of Australia. Family violence in Australia and the National Plan. Canberra: Parliament of Australia (2020). Available online at: https://www.aph.gov.au/Parliamentary_Business/Committees/House/Social_Policy_and_Legal_Affairs/Familyviolence/Report/section?id=committees%2Freportrep%2F024577%2F75208 (accessed 2022, October 20).

Pfitzner N, Fitz-Gibbon K, True J. Responding to the 'shadow pandemic': practitioner views on the nature of and responses to violence against women in Victoria, Australia during the COVID-19 restrictions. Victoria, Australia: Monash University (2020). Available online at: https://apo.org.au/sites/default/files/resource-files/2020-06/apo-nid306064.pdf (accessed 2022, October 20).

Royal Australia and New Zealand College of Obstetricians and Gynaecologists (RANZCOG). COVID-19 Hub. Melbourne: RANZCOG (2020). Available online at: https://ranzcog.edu.au/statements-guidelines/covid-19-statement (accessed 2022, October 20).

Swannel C. Early medical abortion: telehealth restrictions discriminatory. M J Aust. (2020) 21:1. Available online at: https://www.mja.com.au/journal/2020/early-medical-abortion-telehealth-restrictions-discriminatory (accessed 2022, October 20).

Wood D, Griffiths K, Crowley T. Women's work: the impact of the COVID crisis on Australian women. Australia: Grattan Institute (2021). Available online at: https://grattan.edu.au/report/womens-work/ (accessed 2022, October 20).

Zhou N. Female enrolment at Australian universities dropped by 86,000 in 2020 as 'pink recession' hit. The Guardian (2020). Available online at: https://www.theguardian.com/australia-news/2020/nov/12/female-enrolment-at-australian-universities-dropped-by-86000-in-2020-as-pink-recession-hit (accessed 2022, October 20).

Supplementary material. Table S2 Brazil, country case study summary

SDG5	Impact	Action	Future Policy
**Maternity care/ reproductive health**			
Summary	essential services were kept open but with limited access; strong restrictions to prevention and support services; new digital services; 40% increase in maternal deaths in 2020/21 compared to previous year	none; a general trend of decreasing healthcare budgets since 2015 exacerbated the impact of COVID-19	maintenance of essential maternity care services during health crises
Maternity care	limited access to/ accessibility of services due to pandemic restrictions and re-allocation of HCWs; new digital service delivery	none except new digital services; service restrictions due to COVID-19 prioritisation	maintenance of primary and specialised services during health crises
Reproductive services	limited access to services due to pandemic restrictions and move of HCWs; new digital services	none, except new digital services; services reduced due to COVID-19 prioritisation	none
Reproductive rights	no public debate; no explicit restrictions	none; support of maintenance of legal abortion by feminist initiatives, including moving service to digital offers	none
Care for pregnant women/ vaccination	inclusion in vaccination programs, but too late	initial prioritisation of all pregnant women suspended by Ministry of Health in May 2021 and restricted to proof of co-morbidities; criteria of the Operational Plan for comorbidities were very strict and did not cover all situations of gestational risk	none
Health labour market and employment	strong shortage of HCWs in primary and specialised care regardless due to prioritisation of COVID-19 services; higher infection risk of HCWs providing COVID-19 care	none	none
Social effects/ inequalities	increase in social inequalities; maternal death rate was higher among low-income and black women; new digital services may exacerbate inequalities	none; efforts of civil society groups to raise awareness	none
**Gender-based violence**			
Summary	increase of vulnerability but underreporting; reduced opportunity to report; closure of public defenders' offices and transformation to digital services; decrease of 14% in denounces in 2020; strong regional variety; lack of data	intention to create a National Plan against Femicide (Dec. 2020) by federal Government but no signs of implementation; closure of specialised services; reduced collection of data; efforts by municipalities, NGOs and private actors to improve sensitivity and support services	none

Access to services	women's policy stations remained open but limited opportunity to report violence; strong restrictions on access to all services; new digital services; digital provision reduced access of women who lived with the aggressor; services strongly decentralised with high variety and regional differences.	none on national level; efforts by some municipalities, NGOs and private companies to bypass national policy, improve reporting (e.g. digital) and provide services; support by global NGOs	none
Scaling-up/ new programs and training programs	strong limitations due to pandemic priorities and restrictions; strong regional variation; some transformation to digital services	none on national level; some efforts by municipalities, NGOs and private companies to mitigate the problem, keep services open and introduce new programs, including digital offers; some support by global NGOs (e.g. UNICEF)	none
Access of HCWs to help-lines	limited access and longer waiting times; increased need; 30% of HCWs surveyed 2020-21 reported abuse had worsened	none on national level; some municipalities introduced mental health services for HCWs	none
Social effects/ inequalities	increased vulnerability of women living with the aggressor; digitalisation exacerbates social inequalities; lack of data	none	none
**Gender equality**			
Summary	participation of women and female leadership were very poor and weakened; strong political dominance of men; lack of data	none; gender equal goals and monitoring were absent; male dominance in government was strengthened; a National Plan against Feminicide was initiated but not implemented;	none
Women/ female leadership in health policy and COVID-19 governing boards	no; the Bolsonaro government has one of the lowest rates in the history of women ministers; all high-level positions related to the pandemic are held by men	none; all high-level positions related to the pandemic were provided to men; replacements in the Health Ministry were filled by men only; equal opportunity goals not applied to pandemic policy and governance; no monitoring was established	none
Gender equality goals in pandemic policy	none; no prioritisation of all pregnant women in the vaccination calendar, but some municipalities changed the status to priority after strong complaints	none; general lack of pandemic policies and establishment of specific decision-making bodies on national level; some attention on the level of municipalities	none
Gender equality goals in research	None	none, lack of attention and data	none
Access to childcare	closed during 2020/21 and reopened in November 2021	none	none
Access to schools	closed during 2020/21 with online classes and fully reopened in November 2021	none	none
Social effects/ inequalities	increase in intersecting inequalities as women live more often in vulnerable conditions; lack of data	none	none

Source: authors' table, based on expert information and published secondary sources

Key references

Amorim MMR, Takemoto MLS, Fonseca EBD. Maternal deaths with coronavirus disease 2019: a different outcome from low- to middle-resource countries? Am J Obstet Gynecol. (2020) 223(2):298–299. doi:10.1016/j.ajog.2020.04.023

Diniz D, Brito L, Rondon G. Maternal mortality and the lack of women-centered care in Brazil during COVID-19: preliminary findings of a qualitative study. Lancet Regional Health – Americas. (2022) 10:100239. doi:https://doi.org/10-1016/j.lana.2022.100239

Ferigato S, Fernandez M, Amorim M, Ambrogi I, Fernandes LMM, Pacheco R. The Brazilian Government's mistakes in responding to the COVID-19 pandemic. Lancet. (2020) 396(10263):1636. doi:10.1016/S0140-6736(20)32164-4

Fernandez M, Amorim MMR. Morte de grávidas e puérperas por COVID-19. Rede Brasileira de Mulheres Científstas. Nota Técnica (2021) 1. Available online at: https://mulherescientistas.org/wp-content/uploads/2021/05/Nota-Tecnica-n.1-Gravidas-e-Puerperas.pdf (accessed 2022, October 20).

Fórum Brasileiro de Segurança Pública. Violência doméstica durante a pandemia de Covid-19. Forum Brasileiro de Segurança Pública. (2020, April 16). Available online at: https://forumseguranca.org.br/wp-content/uploads/2020/06/violencia-domestica-covid-19-ed02-v5.pdf (accessed 2022, October 20).

Jaccoud L, Sátyro N, Gomes S, Vieira F, Servo L, Fernandez M. Por que a coordenação nacional de políticas públicas importa para os direitos dos cidadãos, especialmente na pandemia? Rede Brasileira de Mulheres Cientístas. Nota Técnica. (2021) 11. Available online at: https://mulherescientistas.org/wp-content/uploads/2021/07/NT-11.pdf (accessed 2022, October 20).

Marques ES, Moraes CL, Hasselman MH, Deslandes SF, Reichenheim MEA. Violência contra mulheres, crianças e adolescentes em tempos de pandemia pela COVID-19: panorama, motivações e formas de enfrentamento. Cad. Saúde Pública. (2021) 36(4):e00074420. https://doi.org/10.1590/0102-311X00074420

Menezes MO, Takemoto MLS, Nakamura-Pereira M, Katz L, Amorim MMR, Salgado HO, et al. Brazilian Group of Studies for COVID-19. Risk factors for adverse outcomes among pregnant and postpartum women with acute respiratory distress syndrome due to COVID-19. Int J Gynaecol Obstet. (2020) 151(3):415–423. doi: 10.1002/ijgo.13407

Silva VR, Ferreira L. Só 55% dos hos dos hospitais que ofereciam serviço de aborto legal no Brasil seguem atendendo na pandemia. Gênero e Número. (2020, July 2). Available online at: http://www.generonumero.media/so-55-dos-hospitais-que-ofereciam-servico-de-aborto-legal-no-brasil-seguem-atendendo-na-pandemia/ (accessed 2022, October 20).

Takemoto MLS, Menezes MO, Andreucci CB, Knobel R, Sousa LAR, Katz L, et al. Maternal mortality and COVID-19. Journal of Maternal-Fetal & Neonatal Medicine. (2020) 16:1–7. doi: 10.1080/14767058.2020.1786056

Wenham C, Fernandez M, Corrêa MG, Lotta G, Schall B, Rocha MC, Pimenta DNG. Gender and race on the frontline: experiences of health workers in Brazil during the COVID-19 pandemic. Social Politics: International Studies in Gender, State & Society. (2021) online. doi:10.1093/sp/jxab031

Zignoni C. O governo que odeia as mulheres: a inércia de Damares Alves na crise do Covid-19. INESC. (2020, April 9). Available online at: https://www.inesc.org.br/o-governo-que-odeia-as-mulheres-a-inercia-de-damares-alves-na-crise-da-codiv-19/ (accessed 2022, October 20).

Supplementary material. Table S3 Germany, country case study summary

SDG5	Impact	Action	Future Policy
**Maternity care/ reproductive health**			
Summary	essential services were kept open; restrictions to prevention and support services; new digital services; some evidence of slightly higher rates of premature births and cesarean deliveries; lack of comprehensive epidemiological data	action was limited to vaccination recommendation and expansion of digital services; in 2022 legal change to de-criminalise physicians' advertisement of early abortion	no attention beyond routine services and existing mandatory legal requirements
Maternity care	access to essential services, but pandemic restrictions (e.g. face mask during birthing; no access of partners; delayed admission in case of COVID-19 infection); local variation; strong restrictions of non-essential services, especially prevention and counselling; digital service provision	new digital services; reimbursement of digital services through social health insurance funds	implementation of digital services in routine care under consideration
Reproductive services	essential services remained open but pandemic restrictions; suspension/ limitations in prevention and support services; staff moved to COVID-19 services; regional/ organisational variation; digital provision	none, except transformation of some services to digital provision	none
Reproductive rights	no restrictions, but negative impact through limited access and availability of services	no change to rights/restrictions, but decriminalisation of physicians' advertisement of early abortion services	none
Care for pregnant women/ vaccination	included in vaccination programs but delayed recommendation by national authorities, also for breast-feeding women	none, except mandatory inclusion of women in vaccine studies; no midwifery experts in pandemic policy	none
Health labour market and employment	high infection risk of midwives; digital services more difficult for self-employed midwives due to high costs and training needs; may reinforce HCW shortage due to drop-outs	none, except mandatory infection prevention law to exclude pregnant midwives and other HCWs from patient care, with full compensation	none
Social inequalities	increased inequalities; increased discrimination; restricted prevention and support services threaten all vulnerable groups (migrants, asylum seekers, ethnic minorities, elderly) most strongly; digitalisation exacerbates inequalities	none; some local variation may apply; some action taken by feminist and migrant networks/ NGOs; no systematic data and monitoring available	none
**Gender-based violence**			
Summary	some attention to the problem; data vary between sources but all show strong increases in all forms of violence; e.g. increase > 100%, during quarantine 7.5% of women/	none; some action taken by NGOs and feminist networks; some media attention but not connected to pandemic policy	none

	10.5% of children experienced sexual violence		
Access to services	routine health and legal services were kept open but prevention services were strongly limited due to lockdown and allocation of staff to pandemic prevention; increase in digital services; NGOs/ feminist groups are important to service providers but with limited resources	no response to new needs; lack of coherent monitoring/ data are scattered and not standardised; feminist activists were not included in pandemic governance	none
Scaling-up/ new programs and training programs	feminist advocacy and some service expansion but no new approaches/ training programs; restrictions due to pandemic policy; regional variation	none	none
Access of HCWs to help-lines	routine services and occupational health services for hospital staff; no specific help-lines for HCWs	none; no support from health professional associations, but some student groups set up online platforms	none
Social inequalities	limited access to services and pandemic policies threaten vulnerable groups most strongly; new need for targeted services for migrants/asylum seekers, minorities; digitalisation worsens inequalities	none	none
**Gender equality**			
Summary	participation of women and female leadership poorly developed and weakened; male scientists more visible in the media; lack of data	none, gender mainstreaming and equal opportunity policy were largely ignored; some sensitivity and more balanced gender composition in new Coalition Government	none, except some general gender equality targets
Women/ female leadership in health policy and COVID-19 governing boards	weak female leadership and participation; no spill-over from female Chancellor (until autumn 2021); women and female groups poorly represented in pandemic policy; no mandatory inclusion of equal opportunity officers in hospital COVID-19 Task Forces; weak media presence of female scientists	none; equal opportunity law not systematically applied to pandemic policy and governance; lack of transparency; no monitoring system; more recently some attention to female COVID-19 scientists	none
Gender equality goals in pandemic policy	largely ignored on all levels	none	none
Gender equality goals in research	lack of data and incentives; little public attention	none, except mandatory inclusion of female participants in vaccine testing	none
Access to childcare	for essential workers/ HCWs, but often strongly limited and not reliable due to staff shortage	None	none
Access to schools	closed during the first wave; re-open with some restrictions/ uncertainties	none, but critical public debate, including attention to increased workload of women	none
Social inequalities	increase in gendered inequalities with strong intersectional effects	lack of political attention	none

Source: authors' table, based on expert information and secondary sources

Key references

Allmendinger J. Kritik am Leopoldina Statement: Das Wohlergehen der Frauen wird nicht adressiert. Tagesspiegel (2020). Available online at: https://www.tagesspiegel.de/wissen/kritik-an-leopoldina-empfehlung-das-wohlergehen-der-frauen-wird-nicht-adressiert/25739444.html (accessed 2022, October 20).

Bariola N, Collins C. The gendered politics of pandemic relief: labor and family policies in Denmark, Germany, and the United States during COVID-19. Am Behav Sci. (2021) 65(12):1671–169. doi:10.1177/00027642211003140

Ciesek, S. Virologie im Medienfokus: Lehren aus der Corona-Krise. Labourjournal. (2022) Juli/August:8–11. Available online at: https://www.laborjournal.de/rubric/essays/essays2022/e22_01.php (accessed 2022, October 20).

Czymara CS, Langenkamp A, Cano T. Cause for concerns: gender inequality in experiencing the COVID-19 lockdown in Germany. European Societies. (2021) 23(Suppl1):S68–81. https://doi.org/10.1080/14616696.2020.1808692

Engelhardt M, Krautstengel A, Patzelt L, Gaudion M, Kamhiye J, Borde T. Auswirkungen der Covid-19 Pandemie auf die Versorgungssituation von geflüchteten Frauen während Schwangerschaft und Geburt. Z Geburtshilfe Neonat. (2021) 225(S 01):P141. doi:10.1055/s-0041-1739903

Pro Familia Medizin. Corona Krise: Reproductive Health Facts – ein Zwischenstand. (2020) Nr 1:1–16. Available online at: https://www.profamilia.de/fileadmin/dateien/fachpersonal/familienplanungsrundbrief/pro_familia_medizin_1-2020.pdf (accessed 2022, October 20).

Hagenbeck C, Pecks U, Fehm T, Borgmeier F, Schleußner E, Zöllkau J. Pregnancy, birth, and puerperium with SARS-CoV-2 and COVID-19. Gynäkologe. (2020) 53(9):614–623. https://doi.org/10.1007/S00129-020-04637-9/TABLES/2

Hipp L, Bünning M. Parenthood as a driver of increased gender inequality during COVID-19? Exploratory evidence from Germany. European Societies. (2021) 23(S1):S658–S673. https://doi.org/10.1080/14616696.2020.1833229

Schmiedhofer M, Derksen C, Dietl JE, Häussler F, Louwen F, Hüner B, et al. Birthing under the condition of the COVID-19 pandemic in Germany: interviews with mothers, partners, and obstetric health care workers. Int. J. Environ. Res. Public Health. (2020) 19*(*3):1486. https://doi.org/10.3390/ijerph19031486

Steiner J, Ebert C. Tatort Wohnzimmer: Gewalt gegen Frauen und Kinder im Corona Lockdown. Ärztetag Podcast.(2020). Available online at: https://www.aerztezeitung.de/Podcasts/Tatort-Wohnzimmer-Gewalt-gegen-Kinder-und-Frauen-im-Corona-Lockdown-410287.html (accessed October 20, 2022).

Zoch G, Bächmann A-C, Vicari B. Care arrangements and parental well-being during the COVID-19 pandemic in Germany. LIfBi Working Paper No 91. Leibniz Institute for Educational Trajectories. (2020). Available online at: https://doi.org/10.5157/LIfBi:WP91:2.0 (accessed 2022, October 20).

Supplementary material. Table S4 United Kingdom, country case study summary

SDG5	Impact	Action	Future Policy
**Maternity care/ reproductive health**			
Summary	essential services remained open but limited access and some replacement by digital provision; strong limitations in non-essential and support services; increase in social inequalities	maternity care was defined as an essential service but restrictions due to pandemic policies and lack of resources; no prioritisation of reproductive health; some action by NHS England to support mothers from minority groups; variation due to devolution	none
Maternity care	essential services remained open but access was limited; strong limitations in support service; limited partner support; new digital services	maternity care was defined as an essential service but lack of attention and resources	none
Reproductive services	strong limitations in provision and access to services due to pandemic restrictions and closure of services; new digital services	no prioritisation of services; introduction of new digital services in NHS services; in March 2020 temporary support of abortion services through availability of early abortion pills without medical supervision	none
Reproductive rights	temporary legal change	temporary legalisation (March 2020) of at-home administration of early abortion pills, previously only available for second pill	permanent availability of at-home administration in England/ Wales; Scotland to decide
Care for pregnant women/ vaccination	inclusion in vaccination programs	none, except vaccination recommendation	none
Health labour market and employment	higher infection risk of HCWs providing COVID-19 care; poor protection and poor worker rights of pregnant HCWs	lack of full legal protection; risk assessment of pregnant women by employer introduced	none
Social inequalities	increase in social inequalities, especially affecting minority and vulnerable groups	none; some action by NHS England to support pregnant women from minority groups	none
**Gender-based violence**			
Summary	strong increase of sexual violence; some expansion of services and digital provision, but limited access due to pandemic restrictions	increased budgets/public funding of services; new law in 2021 to better protect survivors; introduction of digital services	none
Access to services	services were kept open or provided digital, but limitations due to COVID-19 restrictions	increased budgets and public funding of services; improved financial support for NGOs/Charities providing services; digital service	none

		provision; expansion of access through inclusion of pharmacists	
Scaling-up/ new programs and training programs	some expansion of services; some new programs; inclusion of new professional groups; no new training schemes	expansion of providers; new emergency program ‘Ask for ANI’ (Action Needed Immediately) in collaboration with pharmacists; launch of public awareness campaign; Domestic Abuse Act 2021 aims to better protect survivors and address the behaviour of perpetrators	none
Access of HCWs to help-lines	no specific program	none	none
Social inequalities	lack of data, but increased disadvantage of minorities and vulnerable groups, especially through digital services	none	none
**Gender equality**			
Summary	participation of women was limited and female leadership lacking; lack of attention to gender equality goals; increase in intersecting inequalities	none	Scotland to embed the UN CEDAW convention into Scots Law
Women/ female leadership in health policy and COVID-19 governing boards	marginalisation of women in high-level media events; in 2020 43% of daily COVID-19 press conferences featured only male politicians and experts; participation in the Scientific Advisory Group of Experts (SAGE) ranged between 33%-44% and was nearly balanced 2021, but no gender advisor was included	none; equal opportunity goals were not applied to pandemic policy	none
Gender equality goals in pandemic policy	lack of attention	none, except inclusion of COVID-19 vaccination in pregnancy monitoring and attention to men’s higher risk of severe COVID-19 disease; worsening legal conditions and data through suspension of gender pay gap reporting for employers	none
Gender equality goals in research	None	none	none
Access to childcare	facilities were closed during lockdowns; open for essential workers including HCWs	none	None
Access to schools	Schools were closed during first lockdown; open for essential workers including HCWs; remained open for all during second lockdown	none	none
Social inequalities	increase in intersecting inequalities affecting ethnic minorities and vulnerable groups most strongly; digital service provision worsens inequalities; career disadvantages	none	none

	of women with childcare responsibilities		

Source: authors' table, based on expert information and secondary sources

Key references

Bradbury-Jones C, Isham L. The pandemic paradox: the consequences of COVID-19 on domestic violence. J Clin Nurs. (2020) 29:2047–2049. doi:10.1111/jocn.15296

Herten-Crabb A, Wenham C. "I was facilitating everybody else's life. And mine had just ground to a halt": the COVID-19 pandemic and its impact on women in the United Kingdom. Social Politics: International Studies in Gender, State & Society. (2022) online. https://doi.org/10.1093/sp/jxac006

Iacobucci G. Covid-19: Female NHS and care staff report deteriorating health because of pandemic. BMJ (2021) 373:n1157. https://doi.org/10.1136/bmj.n1157

Jardine J, Relph S, Magee L, von Dadelszen P, Morris E, Ross-Davie M, et al. Maternity services in the UK during the coronavirus disease 2019 pandemic: a national survey of modifications to standard care. BJOG: An International Journal of Obstetrics & Gynaecology. (2021) 128(5):880–889. https://doi.org/10.1111/1471-0528.16547

Karavadra B, Stockl A, Prosser-Snelling E, Simpson P, Morris E. Women's perceptions of COVID-19 and their healthcare experiences: a qualitative thematic analysis of a national survey of pregnant women in the United Kingdom. BMC Pregnancy and Childbirth. (2020) 20(1):600. https://doi.org/10.1186/s12884-020-03283-2

Kourti A, Stavridou A, Panagouli E, Psaltopoulou T, Spiliopoulou C, Tsolia M, et al. Domestic violence during the COVID-19 pandemic: a systematic review. Trauma Violence Abuse. (2021) online. https://doi.org/10.1177/15248380211038690

Mansour D. Maintaining sexual and reproductive health services in the UK during COVID-19. BMJ Sex Reprod Health. (2021) 47(4):235–237. https://doi.org/10.1136/bmjsrh-2021-201142

Quach G. Only two UK Covid briefings were led by a female MP, report finds. The Guardian. (2022, January 21). Available online at: https://www.theguardian.com/world/2022/jan/21/only-two-uk-covid-briefings-female-mp-2022-sex-and-power-index (accessed 2022, October 20).

Strauss C, Patel-Campbell C. May 2021 update: COVID-19 and the female health and care workforce survey. London: NHS Confederation. (2021). Available online at: https://www.nhsconfed.org/publications/may-2021-update-covid-19-and-female-health-and-care-workforce-survey (accessed 2022, October 20).

UK Parliament. (2021, May 11). Domestic abuse and COVID-19: a year into the pandemic. Available online at: https://commonslibrary.parliament.uk/domestic-abuse-and-covid-19-a-year-into-the-pandemic/ (accessed 2023, January 4).

UK Government. (2021, January 14). Pharmacies launch codeword scheme to offer 'lifeline' to domestic abuse victims. London: GOV.UK. Available online at: https://www.gov.uk/government/news/pharmacies-launch-codeword-scheme-to-offer-lifeline-to-domestic-abuse-victims (accessed 2022, October 20).

UK Women's Budget Group. One year on: women are less likely than men to feel the Government's response to Covid-19 has met their needs. (2021, March 31). Available online at: https://wbg.org.uk/analysis/reports/one-year-on-women-are-less-likely-than-men-to-feel-the-governments-response-to-covid-19-has-met-their-needs/ (accessed 2022, October 20).

Supplementary material. Table S5 USA, country case study summary

SDG5	Impact	Action	Future Policy
**Maternity care/ reproductive health**			
Summary	decrease of prenatal care visits; increase in demand for mental healthcare; increase in maternal mortality rate (23.8 per 100,000 live births); access to maternity care services was significantly limited but partly replaced by digital services	new digital services were included in COVID-19 emergency policies, but strong variation	extension of emergency digital maternity care and some permanent provision, but high variation
Maternity care	significantly limited access to services and reduced accessibility due to a general decrease and pandemic restrictions; COVID-19 restrictions strongly weaken social/ partner support and support of doulas; new digital service delivery	significant cuts in services due to COVID-19 policy; new digital services were introduced in most insurance programs (e.g. audio- calls combined with monitoring and health coaching); high variation	extension of emergency digital maternity care and some permanent provision; high variation
Reproductive services	limited access to services due to pandemic restrictions and closure of services; new digital services	no prioritisation of services or piecemeal implementation in state-led emergency responses; new digital services but lack of infrastructures	Department of Health and Human Services established Reproductive Healthcare Access Task Force in 2022
Reproductive rights	strong limitations or even suspension of abortion rights through both limited access to services and new legal decisions/ suspension of Roe vs Wade; high variation between states	14 states suspended abortion, only 12 states explicitly protected abortion; medication abortion facilitated by telemedicine is prohibited in 18 states; some attempts to deny Medicaid coverage for certain methods of contraception; high variation between states	suspension of abortion through High Court decision but efforts to protect abortion rights
Care for pregnant women/ vaccination	inclusion in vaccination programs, but lack of attention to the needs of COVID-19 positive women	tailored messaging to pregnant and breast-feeding women to promote vaccination	none
Health labour market and employment	higher infection risk of HCWs providing COVID-19 care; some task-shifting to doulas to mitigate cuts in maternity care provision	none	none
Social inequalities	increase in social inequalities; disruption of reproductive services hit vulnerable populations most strongly; restrictions of Medicaid programs most strongly affected low income people; delays or cancellation of reproductive services were higher in Black and LGBTQIA populations; digital services disadvantaged vulnerable group	none; digital services were thought to reduce racial disparities but the overall effects are less clear and may reinforce inequalities	none
**Gender-based violence**			

Summary	strong increase of sexual violence while access to services was significantly limited; some underreporting due to closed services; early in the pandemic minors made up half of visitors to national sexual assault hotline	none	none
Access to services	strong limitations; one-third of women reported difficulty accessing resources after violence incidents (2020); reduced access to legal services due to courts being closed	some digital service provision	none
Scaling-up/ new programs and training programs	some new programs; some exemption from closures during lockdown; strong regional variation; some transformation to digital services; no new training programs	21 states enacted protections for violence survivors; 5 states included explicit exemptions from non-essential business closures for providers; feminist NGOs increased support services	none
Access of HCWs to help-lines	no information on specific programs	none	none
Social inequalities	inequalities exacerbated for under-served and vulnerable populations; lack of data	none	none
**Gender equality**			
Summary	participation of women and female leadership were overall poor; some recent increase in numbers; lack of attention to gender equality goals; lack of data	none	none
Women/ female leadership in health policy and COVID-19 governing boards	no signs of high-level female leadership but quotas may be higher at lower levels; women accounted for 10% of White House Coronavirus Task Force, but for 84% of staff of Centres for Disease Prevention and Control; data are overall poor	none; equal opportunity goals were not applied to pandemic policy; some increase in female and minority participation in the new Government (2022)	none
Gender equality goals in pandemic policy	lack of attention	none	none
Gender equality goals in research	None	none; a criticism of COVID-19 emergency policies	none
Access to childcare	strong restrictions and some closures; some HCWs lost childcare support affecting women most strongly; high variation	none	none
Access to schools	schools were closed; after re-opening were only small numbers of students accepted; some exceptional openings for HCWs but strong variation	none	none
Social inequalities	increase in gender inequalities most strongly in vulnerable populations; digitalisation worsens inequalities	none	none

Source: authors' table, based on expert information and secondary sources

Key references

Adams C. Pregnancy and birth in the United States during the COVID-19 pandemic: The views of doulas. Birth. (2020) 49(1):116–122. https://doi.org/10.1111/birt.12580

Ahlers-Schmidt CR, Hervey AM, Neil T, Kuhlmann S, Kuhlmann Z. Concerns of women regarding pregnancy and childbirth during the COVID-19 pandemic. Patient Educ Couns. (2020) 103(12):2578–2582. https://doi.org/10.1016/j.pec.2020.09.031

Aiken ARA, Starling JE,Gomperts R, Tec M, Scott JG, Aiken CE. Demand for self-managed online telemedicine abortion in the United States during the Coronavirus disease 2019 (COVID-19) pandemic. Obstet Gynecol. (2020) 136(4):835–837. https://doi.org/10.1097/AOG.0000000000004081

Connor J, Madhavan S, Mokashi M, Amanuel H, Johnson NR, Pace LE, Bartz D. Health risks and outcomes that disproportionately affect women during the Covid-19 pandemic: a review. Soc Sci Med. (2020) 266:113364. https://doi.org/10.1016/j.socscimed.2020.113364

Flor LS, Friedman J, Spencer CN, Cagney J, Arrieta A, Herbert ME, et al. Quantifying the effects of the COVID-19 pandemic on gender equality on health, social, and economic indicators: a comprehensive review of data from March, 2020, to September, 2021. Lancet. (2022) 399:P2381–2397. https://doi.org/10.1016/S0140-6736(22)00008-3

Gutschow K, Davis-Floyd R. The impacts of COVID-19 on US maternity care practices: a follow-up study. Frontiers in Sociology. (2021) 6:655401. https://doi.org/10.3389/fsoc.2021.655401

Maier M, Samari G, Ostrowski J, Bencomo C, McGovern T. ‘Scrambling to figure out what to do’: a mixed method analysis of COVID-19’s impact on sexual and reproductive health and rights in the United States. BMJ Sex Reprod Health. (2021) 47(4):e16. http://dx.doi.org/10.1136/bmjsrh-2021-201081

Robinson LJ, Engelson BJ, Hayes SN. Who is caring for health care workers' families amid COVID-19? Acad Med. (2021) 96(9):1254–1258. https://doi.org/10.1097/ACM.0000000000004022

Sapire R, Ostrowski J, Maier M, Samari G, Bencomo C, McGovern T. COVID-19 and gender-based violence service provision in the United States. PLoS ONE. (2022) 17(2):e0263970. https://doi.org/10.1371/journal.pone.0263970

Stratton P, Gorodetsky E, Clayton J. Pregnant in the United States in the COVID-19 pandemic: a collision of crises we cannot ignore. J Natl Med Assoc. (2021) 113(5):499–503. https://doi.org/10.1016/j.jnma.2021.03.008

Voth Schrag RJ, Leat S, Backes B, Childress S, Wood L. ‘So many extra safety layers’: virtual service provision and implementing social distancing in interpersonal violence service agencies during COVID-19. J Fam Violence. (2022) online. https://doi.org/10.1007/s10896-021-00350-w
